# Peripheral NK cell count predicts response and prognosis in breast cancer patients underwent neoadjuvant chemotherapy

**DOI:** 10.3389/fimmu.2024.1437193

**Published:** 2024-09-30

**Authors:** Chao Zhang, Fengjia Wu, Xiuqing Lu, Sifen Wang, Minqing Wu, Nian Chen, Shanji Fan, Weidong Wei

**Affiliations:** ^1^ State Key Laboratory of Oncology in South China, Guangdong Provincial Clinical Research Center for Cancer, Sun Yat-Sen University Cancer Center, Guangzhou, China; ^2^ The First Affiliated Hospital, Hengyang Medical School, University of South China, Hengyang, China

**Keywords:** peripheral NK cell count, breast cancer, neoadjuvant chemotherapy, nomograms, survival

## Abstract

**Purpose:**

The count of lymphocyte subsets in blood can reflect the immune status of the body which is closely related to the tumor immune microenvironment and the efficacy of NAT. This study aims to explore the relationship between peripheral blood lymphocyte subsets and the efficacy and prognosis of NAT in breast cancer.

**Methods:**

We retrospectively analyzed clinicopathological information and peripheral blood lymphocyte subpopulation counts of patients receiving NAT from January 2015 to November 2021 at Sun Yat-sen University Cancer Center. Kaplan-Meier curves were used to estimate the survival probability. The independent predictors of NAT response and survival prognosis were respectively analyzed by multivariate logistic regression and Cox regression, and nomograms were constructed accordingly. The prediction efficiency of three nomograms was validated separately in the training cohort and the testing cohort.

**Results:**

230 patients were included in the study, consisting of 161 in the training cohort and 69 in the testing cohort. After a median follow-up of 1238 days, patients with higher NK cell value showed higher pCR rates and higher OS and RFS after NAT (all *P* < 0.001). Multivariate analyses suggested NK cell count was an independent predictor of NAT response, OS and RFS. We then built nomograms accordingly and validated the prediction performance in the testing cohort (C index for NAT response: 0.786; for OS: 0.877, for RFS: 0.794).

**Conclusion:**

Peripheral blood NK cell count is a potential predictive marker for BC patients receiving NAT. Nomograms based on it might help predict NAT response and prognosis in BC.

## Introduction

Breast cancer (BC) has become the world’s highest incidence of malignant tumors (1). Among the more than two million new cases each year, almost half of patients eligible for recommended neoadjuvant chemotherapy (NAT) included advanced patients who missed the optimal time for surgery, HER2-positive breast cancer, triple-negative breast cancer, and patients who desired maximum breast preservation during surgery (2, 3). For these patients, NAT is currently widely accepted as the preferred treatment.

NAT is a breakthrough achievement in cancer treatment and has been proven to benefit patients in the middle and advanced stages of a variety of malignancies (4–7). Through NAT, the stage and grading of patients can be reduced, and the probability of complete surgical resection can be significantly improved. When patients show a good response or even achieve a pathological complete response (pCR) in NAT, it often predicts a good prognosis (8, 9). Conversely, for some patients who are insensitive to NAT, NAT may pose a risk of further tumor progression and chemotherapy-related side effects (10). At present, NAT has been more and more widely used in BC patients (9, 11), so it is of great significance to find factors that can predict NAT response to improve patient benefit.

In recent years, an increasing number of diseases have been screened or diagnosed by means of “liquid biopsies” (12, 13). By using easily accessible body fluids such as blood, urine, saliva rich in cells or substances as detection indicators, to obtain disease occurrence, development, outcome and other information. Compared with traditional methods such as histopathology, it is inexpensive, non-invasive and has good repeatability (12). Of note, liquid biopsy has made great progress in the early diagnosis, auxiliary typing, prognosis prediction and treatment response prediction of various malignant tumors (13, 14). However, the prognostic indicators of NAT response in BC are still limited.

Previous studies have confirmed that the state of tumor local immune microenvironment is a key factor affecting the tolerance and efficacy of chemotherapy and radiotherapy for malignant tumors (15, 16). However, there is significant spatial heterogeneity of tumor-infiltrating lymphocytes (TILs) (17, 18), which makes direct analysis of intratumor immune cells too complicated. Lymphocyte is one of the most important cell groups in blood. It has many subgroups and widely participates in the immune process (19). It has been reported that lymphocytes in blood can reflect the immune state of the body to a certain extent and are correlated with infiltrated lymphocytes in tumor tissues (20, 21). At the same time, studies have shown that the total number of peripheral blood lymphocytes is related to the efficacy and prognosis of the primary chemotherapy for breast cancer (22, 23). Therefore, we want to further analyze the relationship between different subsets of lymphocytes in peripheral blood and the NAT efficacy and prognosis in BC.

In this study, we explored the correlation between counts of different lymphocyte subsets in peripheral blood and NAT response in breast cancer. Based on these results, we aimed to construct prediction models to predict NAT response and prognosis.

## Methods

### Study design and patient eligibility

This study retrospectively analyzed the relationship between the values of peripheral blood lymphocyte subsets and the efficacy and prognosis of NAT in breast cancer patients receiving neoadjuvant chemotherapy between January 2015 and November 2021 at Sun Yat-sen University Cancer Center (SYSUCC) in Guangzhou, China. The study was approved by the ethics committee of SYSUCC (B2022-039-01). Due to the retrospective nature of the current study and the anonymous processing of patient information, patients’ written informed consent was waived. We kept all personal data confidential and conducted this study in accordance with the Declaration of Helsinki.

The inclusion criteria were as follows. (I) Female patients older than 18 years; (II) Pathologically confirmed invasive breast cancer; (III) Underwent all cycles of NAT; (IV) Lymphocyte subsets were examined before NAT; (V) Available complete clinicopathological data and specific follow-up data. Patients were excluded when they met the following criteria: (I) Previous antitumor therapy including radiotherapy, chemotherapy, surgery, etc; (II) Second tumor or multisystem tumor; (III) Active or chronic infections, blood system diseases, and autoimmune diseases; (IV) Previous use of drugs or health care products that affect immune function.

### Data collection and patients grouping

Clinicopathological information for all patients was obtained from the electronic medical record system of SYSUCC. General information included age, BMI, molecular subtype, TNM stage, Ki-67 and the lymphocyte subsets test results before NAT. Molecular subtype was classified according to puncture histopathological reports. ER or PR positivity was defined as an immunohistochemical positive rate > 1% (24), and HER2 positivity was determined when the immunohistochemical positive rate was greater than 3+ or 2+ but ERBB2 gene amplification was detected with fluorescence *in situ* hybridization (FISH).

Lymphocyte subsets test is a peripheral blood test in our hospital based on flow cytometry (#NAVIOS, Beckman) which can be used to evaluate the immune status of patients. It is non-invasive, inexpensive and reproducible. All patients received the test within two weeks prior to the first course of NAT, and all patients had their blood taken between 8 a.m. and 12 a.m. It contains counts in the peripheral blood of CD3+ cells (total T cells), CD3+CD4+ cells (CD4+T cells), CD3+CD8+ cells (CD8+T cells), CD19+ cells (total B cells), CD3-CD16+CD56+ cells (NK cells), CD4+CD25+ cells (CD4+ Treg cells) and CD8+CD25+ cells (CD8+ Treg cells). Based on the range of normal reference values given for each subitem of the test, we found that the number of patients below or above the reference value was small, so we chose the median as the cutoff value for the lymphocyte subsets in each group. Age, Ki-67 (25), and TNM staging (26) we referred to the grouping methods and cut-off values of previous studies. As for BMI, considering the height and weight characteristics of the Chinese female population, we refer to the truncation values reported in some Chinese studies (27). Finally, the training cohort and validation cohort were randomly split using R software in a 7:3 ratio.

### Follow and endpoints

Follow-up data were obtained by electronic outpatient records or telephone interviews of SYSUCC. Patients were evaluated every 3 months during NAT, then every 6 months until 5 years, and then annually thereafter. Routine evaluation includes usual hematology and laboratory tests, ultrasound (breast and axillary and neck lymph nodes), or computed tomography. Total body bone imaging is performed annually.

The primary endpoints were overall survival (OS) and relapse-free survival (RFS) which were obtained from the follow-up system. RFS was defined as the time from the end of treatment to the first recurrence of local or regional draining lymph nodes and OS was defined as the time from the start of treatment to death from any cause. Response of NAT was also particularly concerned and it was identified by the postoperative pathological data. Pathological complete response (pCR) was defined as pathological Miller-payne 5 level together with no lymph nodes metastasis after NAT.

### Statistical analysis

The results of lymphocyte subsets were numerical in the preliminary comparative statistics, and were subsequently converted into categorical variables together with age, Ki-67, TNM stage and other variables for subsequent Cox univariate and multivariate regression analysis. The method of converting numerical variables to categorical variables and the cutoff values were described above. The comparison of the means between the two groups was reasonably conducted by using Student’s t-test or Wilcoxon rank sum test according to its normality and homogeneity of variance. The Chi-squared test was used to compare the proportions between the two groups. ROC curve was used to judge the predictive ability of a single indicator to NAT response Survival curves including OS and RFS were estimated using the Kaplan-Meier method and compared with the log-rank test between different groups. Univariate and multivariate Logistic regression analysis of NAT treatment response was performed. Likewise, Univariate and multivariate Cox regression analyses were conducted to explore independent predictors of OS and RFS. Factors were tested according to Schoenfeld residuals, and only factors with a *P* value < 0.05 in the univariate analysis were further included in the multivariate regression analysis. All factors were assessed and reported with their hazard ratios (HRs) and 95% confidence intervals (CIs). We then included statistically significant factors to construct Nomograms for diagnosis and prognosis respectively. Their discriminant efficiency and prediction accuracy were measured by the concordance index (C-index), calibration curves, decision curve analyses (DCA) and time-dependent ROC curves in the training set and validation set. A two-sided *P* value < 0.05 was considered statistically significant. All statistical analysis and result visualization were performed using R software (Version 4.3.1) (stats package, version 4.2.1; car package, version 3.1-0; pROC package, version 1.18.0; ggalluvial package, version 0.12.3; survival package, version 3.3.1; rms package, version 6.4.0; timeROC package, version 0.4; ggplot2 package, version 3.3.6).

## Results

### Characteristics of patients

**Figure 1:**
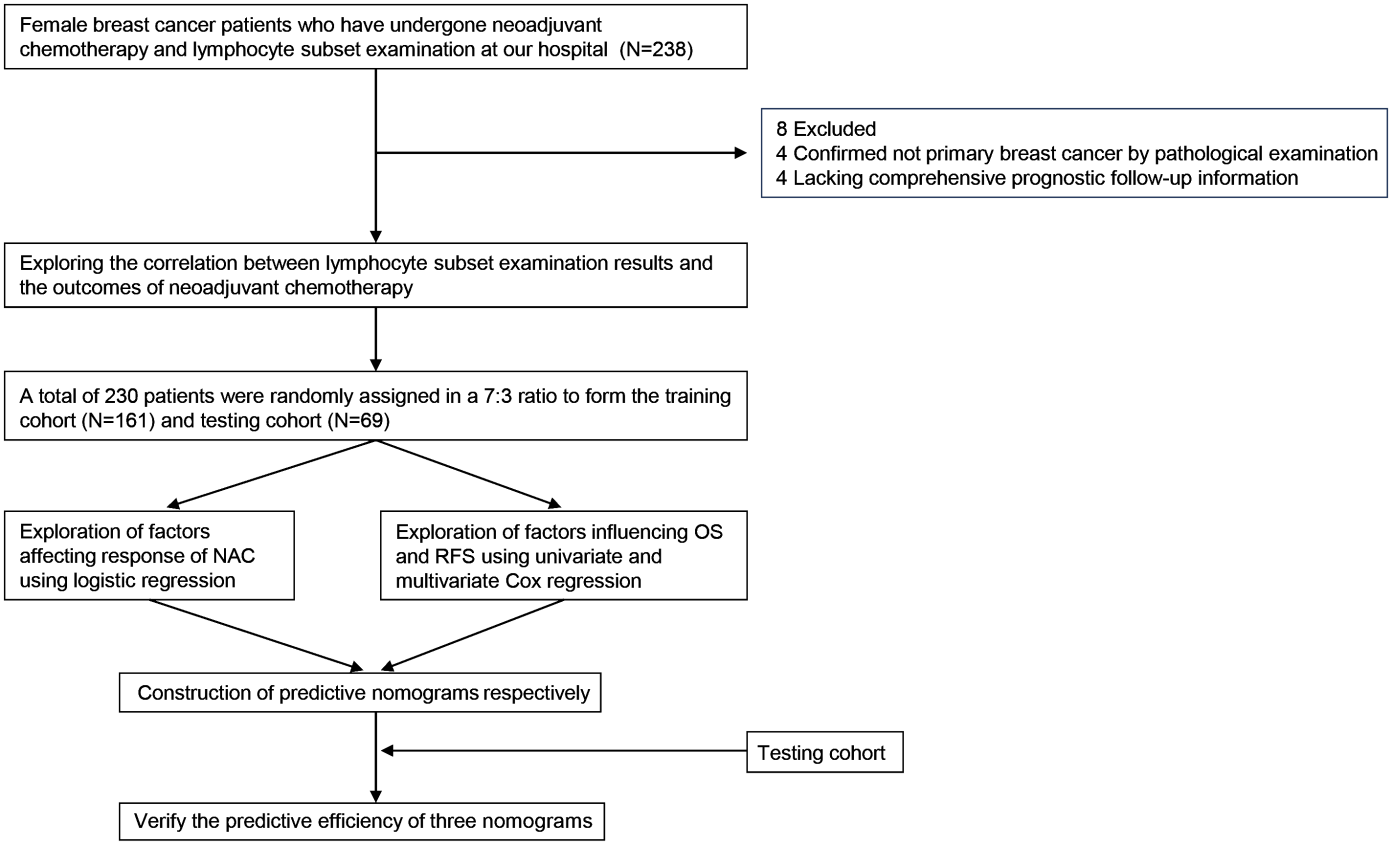
Flowchart of design in this study.

The process of this study was visually presented in a flowchart (**Figure 1**). After excluding 8 patients, including 4 cases where postoperative pathological examination confirmed non-primary breast cancer, and 4 cases with incomplete prognostic follow-up information, a total of 230 patient data were considered valid and included in the analysis of this study. Focusing on the factors related to the efficacy of neoadjuvant therapy in breast cancer patients described above, we first developed a baseline data sheet showing the clinicopathological features of non-pCR versus pCR patients (**Table 1**). The results indicated that there is a statistically significant difference between the two groups in terms of molecular subtype (*P* = 0.010), CD3+CD8+ cells (*P* = 0.042), CD3-CD16+CD56+ cells (*P* < 0.001), CD8+CD25+ cells (*P =* 0.042), T stage (*P* = 0.001), N stage (*P* < 0.001), M stage (*P* = 0.002), recurrence status (*P* < 0.001), and survival status (*P* = 0.001).

**Table 1:** Characteristics of patients in this study.

Characteristics (n%)	non-pCR	pCR	*P* value
N	177 (77.0%)	53 (23%)	
Age, (years)	49 (41-57)	49 (43-55)	0.857
<50	96 (41.7%)	28 (12.2%)	
≥50	81 (35.2%)	25 (10.9%)	
BMI, (kg/m²)			0.853
<24	116 (50.4%)	34 (14.8%)	
≥24	61 (26.5%)	19 (8.3%)	
Ki-67 ^a^			0.482
<30%	73 (31.7%)	19 (8.3%)	
≥30%	104 (45.2%)	34 (14.8%)	
Molecular subtype ^b^			0.010
LumA	40 (17.4%)	14 (6.1%)	
LumB	33 (14.3%)	1 (0.4%)	
Her2	63 (27.4%)	28 (12.2%)	
Basal	41 (17.8%)	10 (4.3%)	
CD3+ ^c^			0.159
<71.5	93 (40.4%)	22 (9.6%)	
≥71.5	84 (36.5%)	31 (13.5%)	
CD3+CD4+ ^c^			0.273
<37.7	92 (40%)	23 (10%)	
≥37.7	85 (37%)	30 (13%)	
CD3+CD8+ ^c^			0.042
<26.85	95 (41.3%)	20 (8.7%)	
≥26.85	82 (35.7%)	33 (14.3%)	
CD19+ ^c^			0.434
<9.25	91 (39.6%)	24 (10.4%)	
≥9.25	86 (37.4%)	29 (12.6%)	
CD3-CD16+CD56+ ^c^			< 0.001
<16.15	107 (46.5%)	8 (3.5%)	
≥16.15	70 (30.4%)	45 (19.6%)	
CD4+CD25+ ^c^			0.434
<19.2	91 (39.6%)	24 (10.4%)	
≥19.2	86 (37.4%)	29 (12.6%)	
CD8+CD25+ ^c^			0.042
<9.1	95 (41.3%)	20 (8.7%)	
≥9.1	82 (35.7%)	33 (14.3%)	
T stage ^d^			0.001
0/1	76 (33%)	39 (17%)	
2	70 (30.4%)	11 (4.8%)	
3	16 (7%)	2 (0.9%)	
4	15 (6.5%)	1 (0.4%)	
N stage ^d^			< 0.001
0	45 (19.6%)	40 (17.4%)	
1	40 (17.4%)	11 (4.8%)	
2	47 (20.4%)	2 (0.9%)	
3	45 (19.6%)	0 (0%)	
M stage ^d^			0.002
0	149 (64.8%)	53 (23%)	
1	28 (12.2%)	0 (0%)	
Relapse status ^e^			< 0.001
0	124 (53.9%)	52 (22.6%)	
1	53 (23%)	1 (0.4%)	
OS status ^e^			0.001
0	141 (61.3%)	52 (22.6%)	
1	36 (15.7%)	1 (0.4%)	

a The Ki-67 index at the diagnosis indicates DNA synthetic activity as measured using immunocytochemistry.

b Molecular subtypes were determined by the expression of ER, PR and HER2.

c The cut-off values were determined as the median for each group.

d Diagnosed based on the AJCC, 2016 criteria (the eighth edition).

e 0 and 1 indicated that the outcome event did not occur and occurred, respectively.

### Examination results of the lymphocyte subpopulations are correlated with the efficacy of NAC

**Figure 2:**
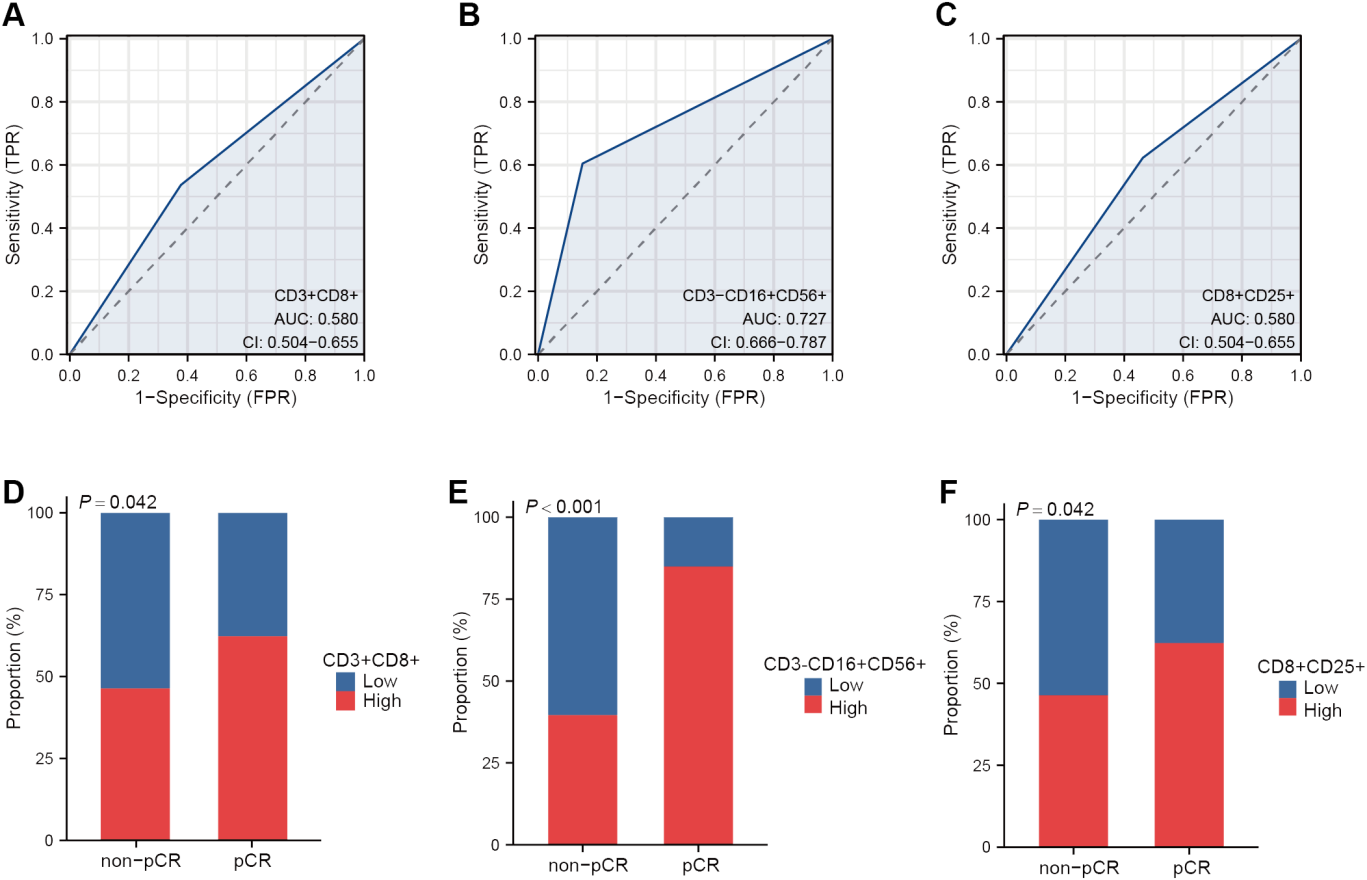
The peripheral lymphocyte subsets correlated with NAT response. ROC curves of CD3+CD8+ cells **(A)**, CD3-CD16+CD56+ cells **(B)** and CD8+CD25+ cells **(C)** in predicting NAT response. Proportion of pCR and non-pCR patients in the high and low group of CD3+ CD8+cells **(D)**, CD3-CD16+CD56+ cells **(E)** and CD8+CD25+ cells **(F)**.

First, ROC curves were used to assess the predictive power of different lymphocyte subsets in the baseline data table for NAT responses. Compared with CD3+CD8+ cells (AUC = 0.580) and CD8+CD25+ cells (AUC = 0.580), CD3-CD16+CD56+ cells had the highest predictive power (AUC = 0.727) (**Figures 2A–C**). Then, the patients were grouped with the median of the above three types of cells as cut-off values. The proportions in the pCR and non-pCR groups show the difference between the groups. CD3-CD16+CD56+ cells had the most significant *P* value (*P* < 0.001) compared with CD3+CD8+ cells (*P* = 0.042) and CD8+CD25+ cells (*P* = 0.042) (**Figures 2D–F**). Taken together, value of CD3-CD16+CD56+ cells had the greatest potential to predict NAT response.

### Survival outcomes in the training cohort

**Figure 3:**
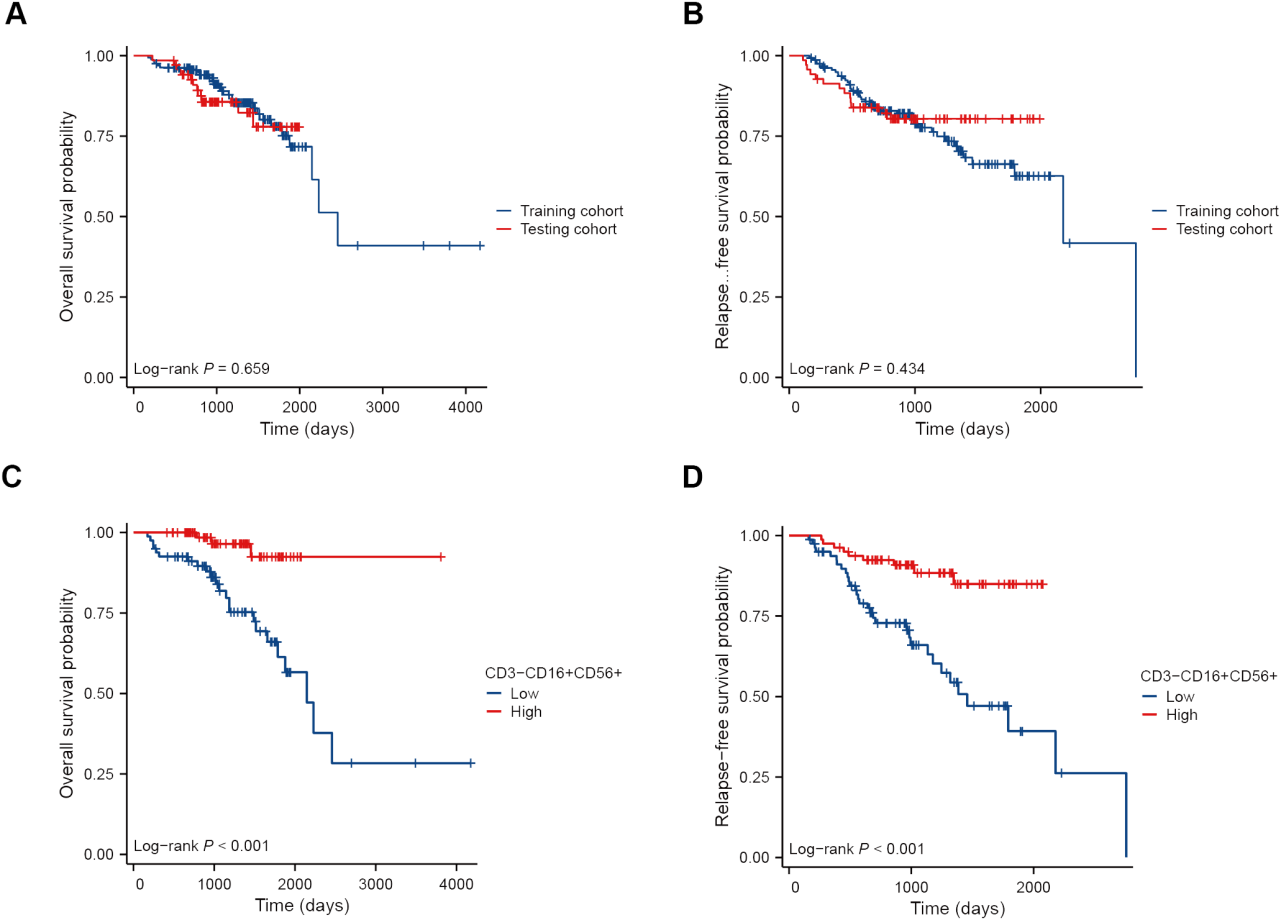
Kaplan-Meier survival curves of BC patients receiving NAT. OS **(A)** and RFS **(B)** curves of patients in the training cohort and the testing cohort. OS **(C)** and RFS **(D)** curves of patients in the high and low NK cell groups.

We randomly allocated 230 breast cancer patients in a 7:3 ratio into the training set (N=161) and validation sets (N=69). The clinical and pathological characteristics of these patients were presented in **Table 2**. The median follow-up time was 1238 days (approximately 41 months) in the training cohort. There was no significant difference in OS and RFS between the training cohort and the testing cohort (both *P* > 0.05) (**Figures 3A, B**). Breast cancer patients with higher levels of CD3-CD16+CD56+ cells in peripheral blood demonstrated significantly improved OS (**Figure 3C**) and RFS compared to those with lower levels (both *P* < 0.001) (**Figure 3D**).

**Table 2:** Characteristics of patients in different cohorts.

Characteristic (%)	All	Training cohort	Testing cohort
	N = 230	N = 161	N = 69
Age at diagnosis (years)		49 (43-57)	47 (41-56)
<50	124 (53.9)	84 (52.2)	40 (58.0)
≥50	106 (46.1)	77 (47.8)	29 (42.0)
BMI (kg/m²)			
<24	150 (65.2)	102 (63.4)	48 (69.6)
≥24	80 (34.8)	59 (36.6)	21 (30.4)
Ki-67 ^a^			
<30%	92 (40.0)	63 (39.1)	29 (42.0)
≥30%	138 (60.0)	98 (60.9)	40 (58.0)
Molecular subtype ^b^			
LumA	54 (23.5)	37 (23.0)	17 (24.6)
LumB	34 (14.8)	26 (16.1)	8 (11.6)
Her2	91 (39.6)	67 (41.6)	24 (34.8)
Basal	51 (22.2)	31 (19.3)	20 (29.0)
NAT response			
non-pCR	177 (77.0)	120 (74.5)	57 (82.6)
pCR	53 (23.0)	41 (25.5)	12 (17.4)
CD3+ (median) ^c^			
<71.5	115 (50.0)	75 (46.6)	40 (58.0)
≥71.5	115 (50.0)	86 (53.4)	29 (42.0)
CD3+CD4+ (median) ^c^			
<37.7	115 (50.0)	74 (46.0)	41 (59.4)
≥37.7	115 (50.0)	87 (54.0)	28 (40.6)
CD3+CD8+ (median) ^c^			
<26.85	115 (50.0)	79 (49.0)	36 (52.2)
≥26.85	115 (50.0)	82 (51.0)	33 (47.8)
CD19+ (median) ^c^			
<9.25	115 (50.0)	81 (50.0)	34 (49.3)
≥9.25	115 (50.0)	80 (50.0)	35 (50.7)
CD3-CD16+CD56+ (median) ^c^			
<16.15	115 (50.0)	81 (50.0)	34 (49.3)
≥16.15	115 (50.0)	80 (50.0)	35 (50.7)
CD4+CD25+ (median) ^c^			
<19.2	115 (50.0)	78 (48.4)	37 (53.6)
≥19.2	115 (50.0)	83 (51.6)	32 (46.4)
CD8+CD25+ (median) ^c^			
<9.1	115 (50.0)	85 (52.8)	30 (43.5)
≥9.1	115 (50.0)	76 (47.2)	39 (56.5)
T stage^d^			
T0&T1	115 (50.0)	82 (50.9)	33 (47.8)
T2	81 (35.2)	54 (33.5)	27 (39.1)
T3	18 (7.8)	14 (8.7)	4 (5.8)
T4	16 (7.0)	11 (6.8)	5 (7.2)
N stage^d^			
N0	85 (37.0)	60 (37.3)	25 (36.2)
N1	51 (22.2)	33 (20.5)	18 (26.1)
N2	49 (21.3)	35 (21.7)	14 (20.3)
N3	45 (19.6)	33 (20.5)	12 (17.4)
M stage^d^			
M0	202 (87.8)	142 (88.2)	60 (87.0)
M1	28 (12.2)	19 (11.8)	9 (13.0)

a The Ki-67 index at the diagnosis indicates DNA synthetic activity as measured using immunocytochemistry.

b Molecular subtypes were determined by the expression of ER, PR and HER2.

c The cut-off values were determined as the median for each group.

d Diagnosed based on the AJCC, 2016 criteria (the eighth edition).

### Establishing and validation a predictive model for NAC response

**Figure 4:**
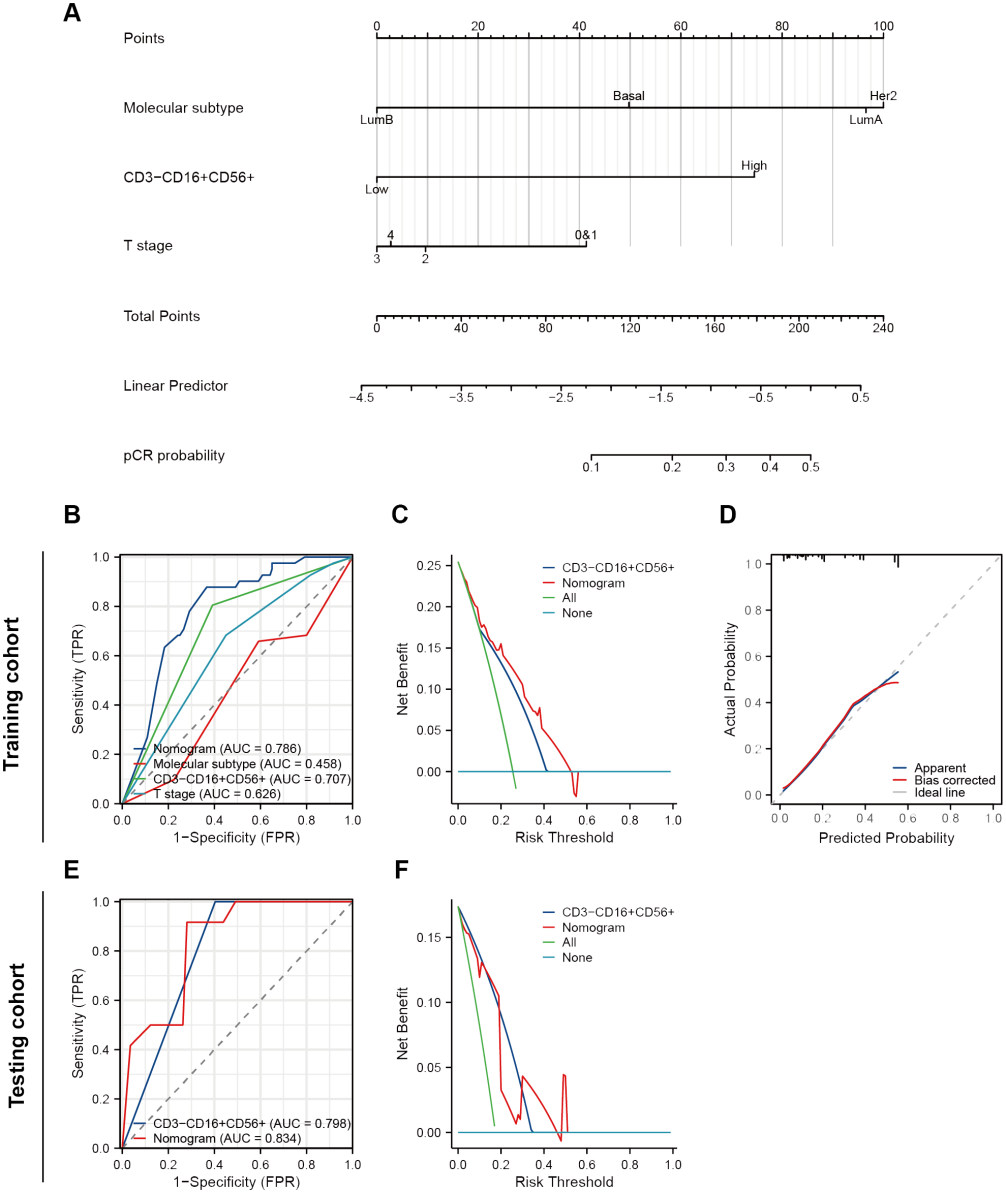
Construction and validation of a nomogram to predict NAT response. **(A)** A nomogram predicting response of NAT. **(B)** ROC curves of different predictors in the training cohort. **(C)** A decision curve analysis of this nomogram in the training cohort. **(D)** The calibration curve of the nomogram. **(E)** ROC curves of different predictors in the testing cohort. **(F)** A decision curve analysis of this nomogram in the testing cohort.

Initially, we conducted univariate and multivariate logistic regression analyses. The results indicated that molecular subtype, CD3-CD16+CD56+ cells, and T stage had statistical significance in the univariate analysis (all *P* < 0.05) (**Table 3**). When considering variables with *P* < 0.05, the multivariate analysis revealed that only CD3-CD16+CD56+ cells remained statistically significant (*P* < 0.001). Considering that an individual variable often has limited predictive power, we included all three variables with *P* < 0.05 in univariate regression analysis to construct the predictive nomogram (**Figure 4A**). The model was satisfactory with a C-index at 0.786 (95% CI 0.711 - 0.862). Subsequently, ROC curves, DCA plots, and calibration curves were employed to assess the predictive performance of the Nomogram model. The Nomogram model exhibited superior predictive performance for NAC response (AUC = 0.786), surpassing the univariate predictive ability of CD3-CD16+CD56+ cells (AUC = 0.707). Furthermore, the Nomogram model yielded greater overall net benefits for breast cancer patients in NAC prediction compared to CD3-CD16+CD56+ cell prediction (**Figures 4B–D**). Consistent findings were obtained in the testing cohort (Nomogram AUC = 0.834, CD3-CD16+CD56+ AUC = 0.798) (**Figures 4E, F**).

**Table 3:** Univariate and multivariate logistic regression analysis of NAT response.

Characteristics	Total (N)	Univariate		Multivariate	
		Odds Radio (95%CI)	*P* value	Odds Radio (95%CI)	*P* value
**Age (years)**	161				
<50	84	Reference			
≥50	77	1.562 (0.765 - 3.189)	0.221		
**BMI (kg/m²)**	161				
<24	102	Reference			
≥24	59	0.997 (0.477 - 2.080)	0.993		
Ki-67 ^a^	161				
<30%	63	Reference			
≥30%	98	0.878 (0.426 - 1.807)	0.723		
Molecular subtype ^b^	161				
LumA	37	Reference			
LumB	26	0.074 (0.009 - 0.609)	0.015 *	0.130 (0.015 - 1.136)	0.065
Her2	67	0.965 (0.416 - 2.241)	0.934	1.076 (0.432 - 2.679)	0.875
Basal	31	0.274 (0.078 - 0.953)	0.042 *	0.372 (0.098 - 1.407)	0.145
CD3+ ^c^	161				
<71.5	75	Reference			
≥71.5	86	1.511 (0.734 - 3.112)	0.263		
CD3-CD16+CD56+ ^c^	161				
<15.6	81	Reference			
≥15.6	80	6.407 (2.725 - 15.063)	< 0.001 *	4.834 (1.961 - 11.915)	< 0.001 *
CD8+CD25+ ^c^	161				
<9.1	85	Reference			
≥9.1	76	1.615 (0.791 - 3.300)	0.188		
T stage ^d^	161				
0&1	82	Reference			
2	54	0.438 (0.192 - 1.000)	0.050 *	0.511 (0.208 - 1.257)	0.144
3	14	0.321 (0.067 - 1.537)	0.155	0.417 (0.078 - 2.231)	0.307
4	11	0.193 (0.023 - 1.583)	0.125	0.442 (0.046 - 4.270)	0.481
M stage ^d^	161				
0	142	Reference			
1	19	0.000 (0.000 - Inf)	0.985		

a The Ki-67 index at the diagnosis indicates DNA synthetic activity as measured using immunocytochemistry.

b Molecular subtypes were determined by the expression of ER, PR and HER2.

c The cut-off values were determined as the median for each group.

d Diagnosed based on the AJCC, 2016 criteria (the eighth edition). *P<0.05.

### Establishment of prognostic prediction models

**Figure 5:**
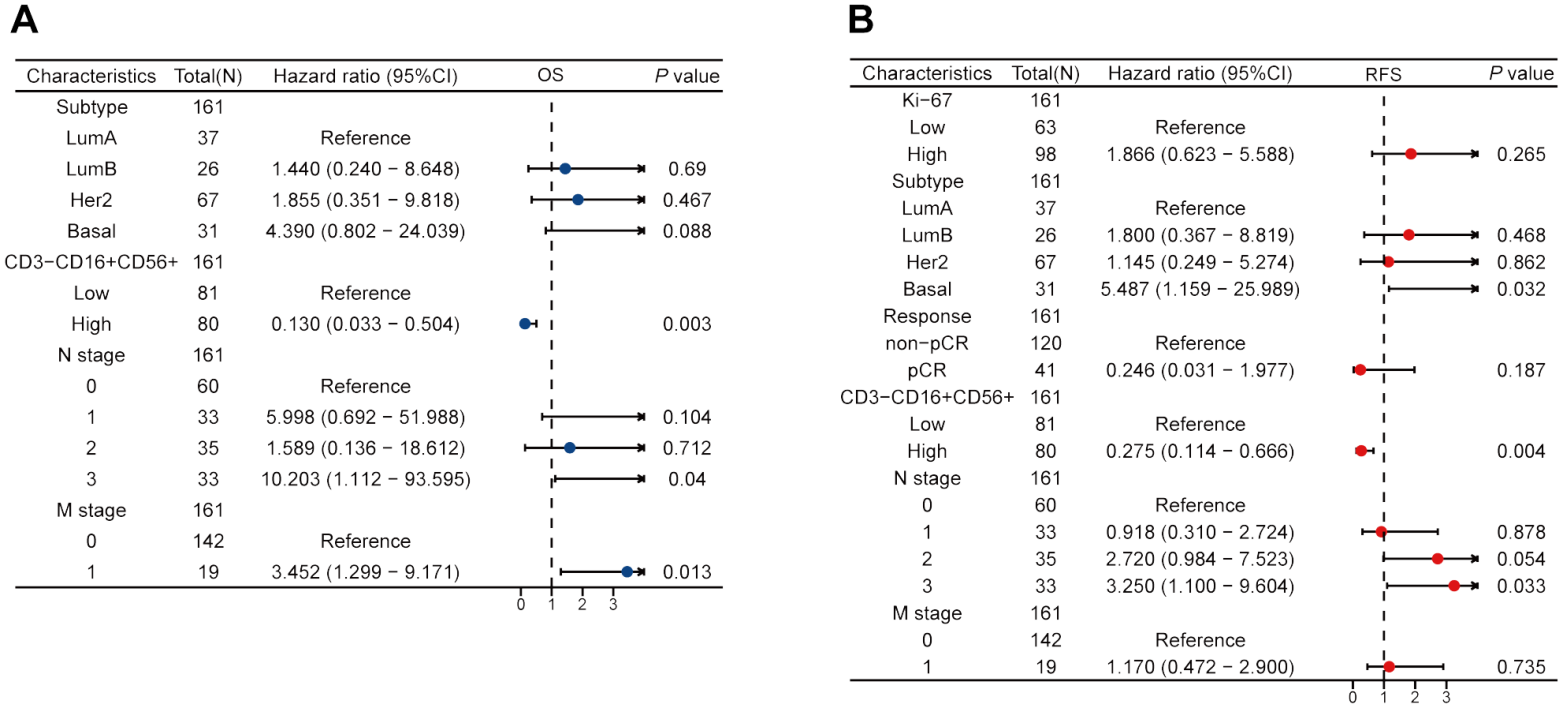
Multivariate Cox regression forest maps of OS **(A)** and RFS **(B)** in the training cohort.

**Figure 6:**
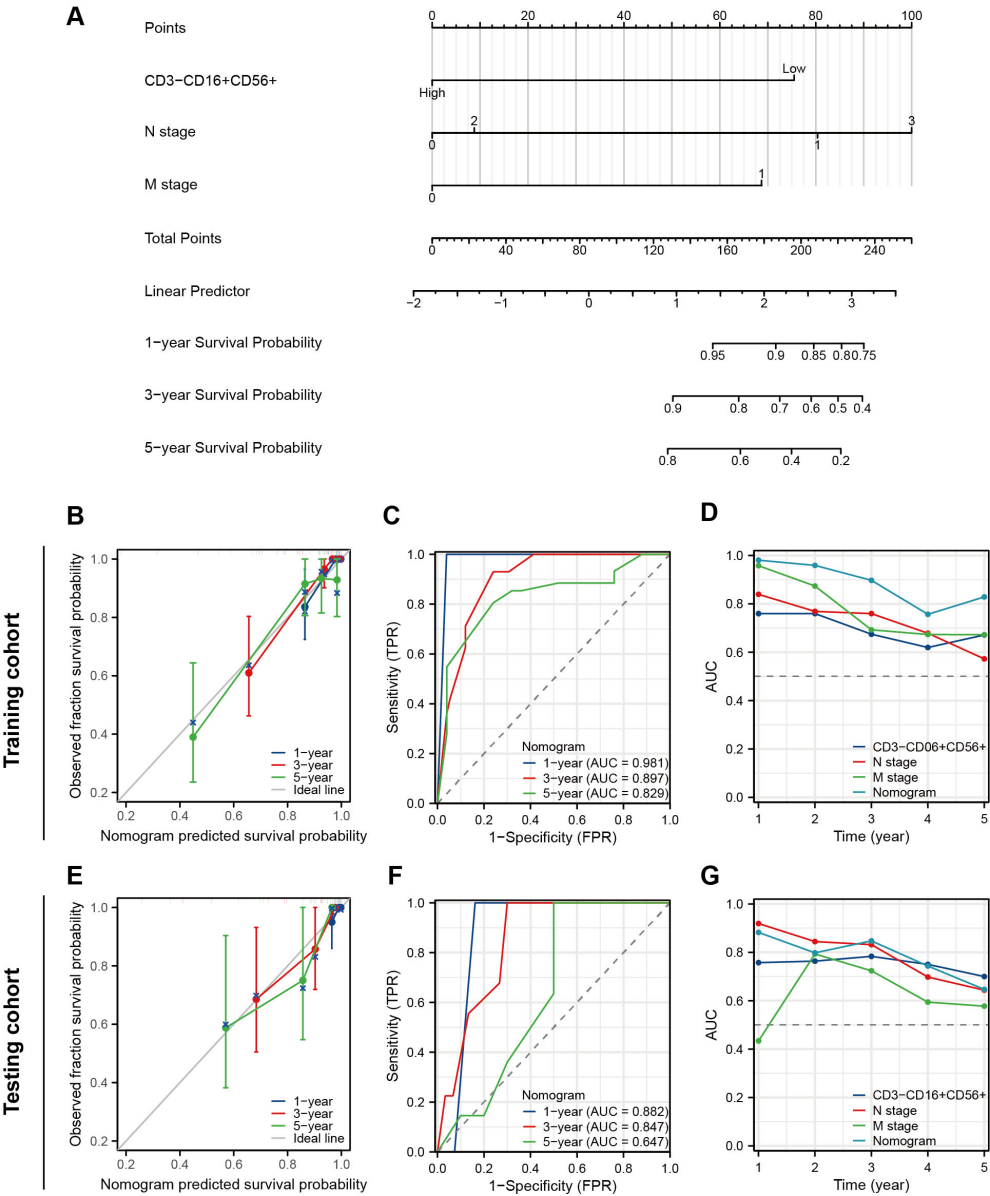
Construction and validation of a nomogram to predict OS. **(A)** A nomogram predicting 1-, 3- and 5-year OS. **(B)** The prognostic calibration curves in the training cohort. **(C)** Time-dependent ROC curves of the nomogram in the training cohort. **(D)** Time-dependent AUC curves for different predictors in the training cohort. **(E)** The prognostic calibration curves in the testing cohort. **(F)** Time-dependent ROC curves of the nomogram in the testing cohort. **(G)** Time-dependent AUC curves for different predictors in the testing cohort.

**Figure 7:**
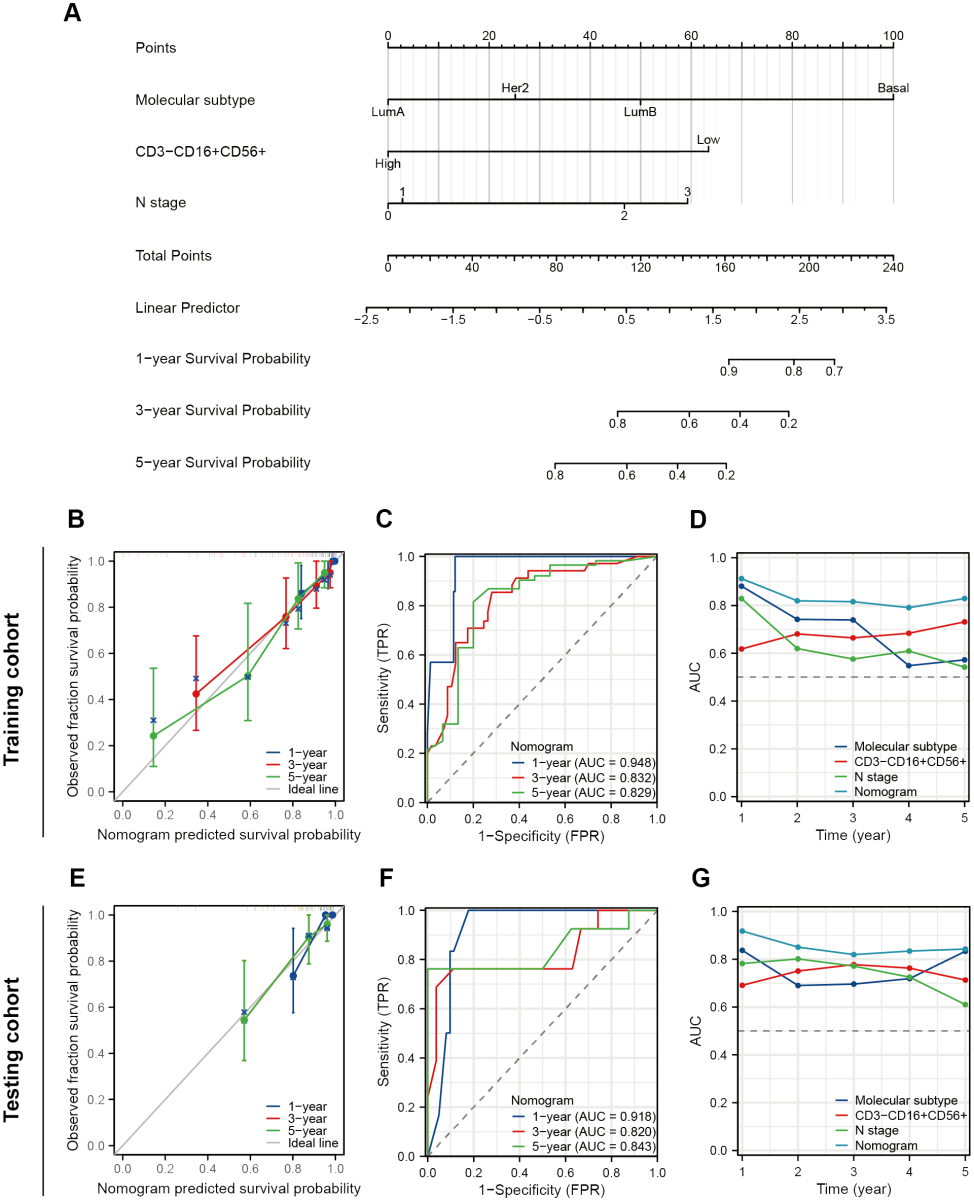
Construction and validation of a nomogram to predict RFS. **(A)** A nomogram predicting 1-, 3- and 5-year RFS. **(B)** The prognostic calibration curves in the training cohort. **(C)** Time-dependent ROC curves of the nomogram in the training cohort. **(D)** Time-dependent AUC curves for different predictors in the training cohort. **(E)** The prognostic calibration curves in the testing cohort. **(F)** Time-dependent ROC curves of the nomogram in the testing cohort. **(G)** Time-dependent AUC curves for different predictors in the testing cohort.

We performed the univariate Cox regression analysis for OS and RFS in the training cohort and presented the results in the table (**Table 4**). The results indicated that molecular subtypes, CD3-CD16+CD56+ cells, T stage, N stage, and M stage were significantly associated with OS in breast cancer patients after neoadjuvant chemotherapy. Furthermore, Ki-67, molecular subtypes, NAC response, CD3-CD16+CD56+ cells, N stage and M stage were significantly associated with RFS. Variables with p-values less than 0.1 in the univariate Cox regression analysis were included in the multivariate Cox regression analysis. CD3-CD16+CD56+ cells, N stage, and M stage were identified as independent prognostic factors for OS, while molecular subtypes, CD3-CD16+CD56+ cells, and N stage were confirmed as independent prognostic factors for RFS (**Figure 5**). Based on the independent prognostic factors obtained from the multivariate Cox regression, nomogram models were constructed to predict Overall Survival (OS) and Recurrence-Free Survival (RFS) respectively (**Figures 6A**, **7A**).

**Table 4:** Univariate Cox regression analysis of OS and RFS.

Characteristics		Overall survival		Relapse-free survival	
		Hazard ratio (95%CI)	*P* value	Hazard ratio (95%CI)	*P* value
Age (years)					
<50		Reference		Reference	
≥50		1.181 (0.545 - 2.557)	0.673	0.529 (0.279 - 1.006)	0.052
BMI (kg/m²)					
<24		Reference		Reference	
≥24		1.154 (0.510 - 2.611)	0.731	1.134 (0.594 - 2.167)	0.703
Ki-67 ^a^					
<30%		Reference		Reference	
≥30%		2.324 (0.922 - 5.860)	0.074	3.040 (1.397 – 6.617)	0.005 *
Molecular subtype^b^					
LumA		Reference		Reference	
LumB		5.656 (1.141 - 28.026)	0.034 *	5.316 (1.700 – 16.625)	0.004 *
Her2		2.731 (0.560 - 13.329)	0.214	1.387 (0.427 – 4.507)	0.586
Basal		8.628 (1.877 - 39.653)	0.006 *	9.039 (2.955 – 27.645)	< 0.001 *
NAC response					
non-pCR		Reference		Reference	
pCR		0.169 (0.023 – 1.260)	0.083	0.069 (0.009 – 0.502)	0.008 *
CD3+ ^c^					
<71.5		Reference		Reference	
≥71.5		1.046 (0.483 - 2.267)	0.909	0.788 (0.420 - 1.479)	0.458
CD3+CD4+ ^c^					
<37.7		Reference		Reference	
≥37.7		1.516 (0.687 - 3.343)	0.303	0.642 (0.342 - 1.206)	0.169
CD3+CD8+ ^c^					
<26.9		Reference		Reference	
≥26.9		0.839 (0.387 - 1.821)	0.657	1.356 (0.722 - 2.548)	0.344
CD19+ ^c^					
<9.3		Reference		Reference	
≥9.3		1.078 (0.497 - 2.338)	0.849	0.645 (0.343 - 1.211)	0.172
CD3-CD16+CD56+ ^c^					
<15.6		Reference		Reference	
≥15.6		0.147 (0.044 - 0.494)	0.002 *	0.237 (0.113 - 0.501)	< 0.001 *
CD4+CD25+ ^c^					
<19.2		Reference		Reference	
≥19.2		0.793 (0.362 - 1.734)	0.561	0.777 (0.413 - 1.461)	0.434
CD8+CD25+ ^c^					
<9.1		Reference		Reference	
≥9.1		0.881 (0.389 - 1.994)	0.762	1.008 (0.535 - 1.900)	0.980
T stage ^d^					
0&1		Reference		Reference	
2		1.391 (0.546 - 3.543)	0.489	1.286 (0.627 - 2.639)	0.492
3		0.830 (0.205 - 3.361)	0.794	2.607 (1.056 – 6.437)	0.038 *
4		3.311 (1.057 - 10.376)	0.040 *	1.204 (0.341 – 4.254)	0.773
N stage ^d^					
0		Reference		Reference	
1		8.977 (1.095 - 73.586)	0.041 *	1.273 (0.440 - 3.678)	0.656
2		3.707 (0.413 - 33.257)	0.242	2.238 (0.910 – 5.503)	0.079
3		16.677 (2.160 - 128.773)	0.007 *	3.550 (1.469 – 8.582)	0.005 *
M stage ^d^					
0		Reference		Reference	
1		7.957 (3.630 - 17.443)	< 0.001 *	3.013 (1.399 - 6.490)	0.005 *

a The Ki-67 index at the diagnosis indicates DNA synthetic activity as measured using immunocytochemistry.

b Molecular subtypes were determined by the expression of ER, PR and HER2.

c The cut-off values were determined as the median for each group.

d Diagnosed based on the AJCC, 2016 criteria (the eighth edition). *P<0.05.

### Assessment of the prognostic performance of the predictive models

The prognostic nomogram exhibited favorable discriminative accuracy and predictive capacity for OS with a C-index at 0.877 (95%CI 0.845-0.908). In the prognostic calibration curves for the training and validation cohorts, the predicted outcomes for 1-, 3-, and 5-year prognosis closely aligned with the ideal line (**Figures 6B, E**). The time-dependent ROC curve demonstrated that the model exhibited high accuracy in predicting patient prognosis, particularly in terms of 1- and 3-year survival rates (1-year, AUC = 0.981 and 0.882; 3-years, AUC = 0.897 and 0.847; 5-years, AUC = 0.829 and 0.647) (**Figures 6C, F**). The time-dependent AUC curve was employed to compare the predictive accuracy of the nomogram model, CD3-CD16+CD56+ cells, N stage, and M stage in terms of prognosis. The results indicated that for the majority of time points within the 0-5 years period, the nomogram exhibits a valuable predictive performance for OS (at any time AUC > 0.65) (**Figures 6D, G**).

As for RFS, the nomogram model demonstrates a more satisfactory predictive accuracy and performance with a C-index at 0.794 (95% CI 0.754-0.833). The calibration plot documented a good agreement between the observed 1-, 3-, and 5-year RFS rates and the nomogram-predicted 1-, 3-, and 5-year OS rates (**Figures 7B, E**). Both the training and testing cohorts demonstrated time-dependent ROC curves with AUC exceeding 0.8 at 1-, 3-, and 5-year intervals (training set 0.948, 0.832, 0.829, testing cohort 0.918, 0.820, 0.843) (**Figures 7C, F**). Furthermore, in the time-dependent AUC curves, the Nomogram exhibited excellent predictive accuracy for RFS at any given time point (AUC > 0.8), surpassing other variables such as molecular subtypes, CD3-CD16+CD56+ cells, and N stage (**Figures 7D, G**). In short, these two nomograms exhibited good efficacy in predicting OS and RFS for patients undergoing NAT.

## Discussion

As the concept of NAT has been gradually accepted, more and more patients with middle and advanced BC have adopted neoadjuvant therapy (2, 28). However, some patients who are not sensitive to chemotherapy do not benefit from NAT and are even at risk for disease progression (29). The response of NAT is closely related to patient prognosis and can even be used as an alternative prognostic endpoint in some clinical studies (9, 30). Therefore, it is of great clinical significance to predict the efficacy of NAT by developing new and easily available indicators.

In this study, we first retrospectively analyzed the correlation between various lymphocyte subsets in peripheral blood and NAT response. We found that T cells, NK cells and CD8+ Treg were higher in the pCR group than in the non-pCR group, and the difference between NK cells was most significant. In addition, patients in the high NK cells group had higher OS and RFS than those in the low NK cells group. Multivariate logistic and Cox regression indicated that peripheral blood NK cell count was an independent predictor of NAT response, OS and RFS. Subsequently, NK cell counts combined with other clinicopathological factors such as molecular typing and TNM staging were incorporated to construct predictive nomograms, which were used to predict NAT response, OS, and RFS, respectively.

Paclitaxel, doxorubicin and cyclophosphamide, which are frequently included in NAT regimens for breast cancer, have been reported to have a synergistic killing effect with anti-tumor immunity (31–33). The immune state of the body and the tumor immune microenvironment greatly affect the efficacy of chemotherapy in diverse ways (34–36). As professional immune cells, lymphocytes play an important role in the innate and cellular immune pathways involved in tumor clearance. Previous studies have shown that lymphocytes can be divided into many subgroups, which usually express different surface markers and perform their specific immune-related functions (37, 38). NK cells, for example, often express CD16 and CD56 surface markers, with their strong immune clearance ability, play a synergistic anti-tumor role in tumor radiotherapy and chemotherapy (39). While common regulatory T cells (Treg), known as CD4+CD25+CD127-/low, are prone to mediate immune suppression by secreting cytokines such as TGF-β (40).

Just as the gradually widely used peripheral blood circulating small molecules such as DNA and non-coding RNA reflect certain characteristics of tumor cells (13), peripheral blood lymphocytes have also been proved to be significantly correlated with lymphocyte infiltration and immune microenvironment in tumor tissues. In a variety of malignancies, high level lymphocytes with immune killing activity in the peripheral blood often suggest abundant immune infiltration in tumor tissue and a good therapeutic effect, while the enrichment of immune inhibitory cells often predicts a poor therapeutic effect and prognosis (41, 42). Joan et al. ‘s study confirmed that tumor infiltrated NK cells (TINK) were an independent predictor of breast cancer NAT response, and higher NK cell infiltration predicted higher pCR probability (*P* < 0.0001) and higher survival (43), which was highly consistent with the results of this study.

Patients with different molecular subtypes have different responses to NAT (44). It has been reported that estrogen receptor or progesterone receptor positive luminal subtype breast cancer has a lower pCR rate in NAT (45), which is also reflected in the results of this study. From this Nomogram, we can see that luminal B subtype corresponded to the lowest contribution score. This suggests that the scope of application of NAT should not be over-enlarged, and clinical practice should be based on guideline recommendations and various clinicopathological characteristics and needs of patients, so as to select the people who are most likely to benefit from NAT.

TNM staging system has been proved to be one of the most valuable prognostic indicators in previous studies (46). The results of this study showed that higher T staging suggested poor NAT response rates and patients with higher N and M staging have worse OS and RFS. It is consistent with the results of previous studies. Notably, NAT response in this study was not an independent predictor of OS or RFS (*P* > 0.05), suggesting that pCR did not translate into a survival advantage during NAT in the patients included in this study. We speculated that the sample size was too small or the follow-up time was not long enough, so the difference in survival of people with different responses to NAT did not reach statistical significance.

However, there are some limitations in this study. First, this study was a single-center retrospective analysis with a small sample size, and the results may be subject to error or bias. Moreover, all of the patients were from China, suggesting that current findings may not be applicable to patients from other geographic regions. Second, the cell count data in this study were directly derived from the results of the lymphocyte examination program in the hospital, and the use of the median as the cut-off value may weaken the representativeness of this indicator. Because the low and high subgroups in this study still include the normal range and that are too low or too high. It is well known that patients with abnormal lymphocyte counts may be in a state of immune abnormality and have some prognostic factors present. Third, NK cells can still be divided into smaller subpopulations with different functions, and CD3-CD16+CD56+ labeling does not perfectly represent the population of NK cells *in vivo*. Fourth, due to the small sample size of the study, we were unable to perform further subgroup analyses based on breast cancer subtypes or different chemotherapy regimens. Therefore, further studies with multi-center and larger sample size need to be conducted to confirm the conclusion of this study.

## Conclusion

Peripheral blood NK cell count is an independent predictor of NAT response in BC patients. On this basis, we constructed and verified nomograms for predicting NAT responses, OS and RFS. All three nomograms showed good predictive power and consistency with actual clinical outcomes.

## Data Availability

The original contributions presented in the study are included in the article/supplementary material. Further inquiries can be directed to the corresponding authors.
